# Disruption of the Basal Body Protein POC1B Results in Autosomal-Recessive Cone-Rod Dystrophy

**DOI:** 10.1016/j.ajhg.2014.06.012

**Published:** 2014-08-07

**Authors:** Susanne Roosing, Ideke J.C. Lamers, Erik de Vrieze, L. Ingeborgh van den Born, Stanley Lambertus, Heleen H. Arts, POC1B Study Group, Karsten Boldt, Elfride de Baere, Caroline C.W. Klaver, Frauke Coppieters, David A. Koolen, Dorien Lugtenberg, Kornelia Neveling, Jeroen van Reeuwijk, Marius Ueffing, Sylvia E.C. van Beersum, Marijke N. Zonneveld-Vrieling, Theo A. Peters, Carel B. Hoyng, Hannie Kremer, Lisette Hetterschijt, Stef J.F. Letteboer, Erwin van Wijk, Ronald Roepman, Anneke I. den Hollander, Frans P.M. Cremers

**Affiliations:** 1 Department of Human Genetics, Radboud University Medical Center, PO Box 9101, 6500 HB Nijmegen, the Netherlands; 2 Radboud Institute for Molecular Life Sciences, Radboud University Nijmegen, PO Box 9101, 6500 HB Nijmegen, the Netherlands; 3 Department of Otorhinolaryngology, Radboud University Medical Center, PO Box 9101, 6500 HB Nijmegen, the Netherlands; 4 The Rotterdam Eye Hospital, PO Box 70030, 3000 LM Rotterdam, the Netherlands; 5 Department of Ophthalmology, Radboud University Medical Center, PO Box 9101, 6500 HB Nijmegen, the Netherlands

## Abstract

Exome sequencing revealed a homozygous missense mutation (c.317C>G [p.Arg106Pro]) in *POC1B*, encoding POC1 centriolar protein B, in three siblings with autosomal-recessive cone dystrophy or cone-rod dystrophy and compound-heterozygous *POC1B* mutations (c.199_201del [p.Gln67del] and c.810+1G>T) in an unrelated person with cone-rod dystrophy. Upon overexpression of *POC1B* in human TERT-immortalized retinal pigment epithelium 1 cells, the encoded wild-type protein localized to the basal body of the primary cilium, whereas this localization was lost for p.Arg106Pro and p.Gln67del variant forms of POC1B. Morpholino-oligonucleotide-induced knockdown of *poc1b* translation in zebrafish resulted in a dose-dependent small-eye phenotype, impaired optokinetic responses, and decreased length of photoreceptor outer segments. These ocular phenotypes could partially be rescued by wild-type human *POC1B* mRNA, but not by c.199_201del and c.317C>G mutant human *POC1B* mRNAs. Yeast two-hybrid screening of a human retinal cDNA library revealed FAM161A as a binary interaction partner of POC1B. This was confirmed in coimmunoprecipitation and colocalization assays, which both showed loss of FAM161A interaction with p.Arg106Pro and p.Gln67del variant forms of POC1B. FAM161A was previously implicated in autosomal-recessive retinitis pigmentosa and shown to be located at the base of the photoreceptor connecting cilium, where it interacts with several other ciliopathy-associated proteins. Altogether, this study demonstrates that *POC1B* mutations result in a defect of the photoreceptor sensory cilium and thus affect cone and rod photoreceptors.

## Introduction

Inherited cone disorders are a heterogeneous group of diseases that primarily affect cone photoreceptors and have an estimated worldwide prevalence of 1:30,000–1:40,000.^1–3^ They can be divided into progressive forms of cone dystrophy (COD) and the more stationary disorders, also named cone-dysfunction syndromes. The stationary subtypes, such as achromatopsia (ACHM), are congenital, and children with ACHM present with congenital nystagmus, significantly reduced visual acuity, severe photophobia, poor or absent color vision, and normal fundi. Electroretinography (ERG) shows no or residual cone responses and normal rod responses. COD, on the other hand, starts in childhood or early adult life and causes progressive deterioration of visual acuity and color vision, as shown by reduced cone responses on ERG.^4,5^ The fundus examination in COD varies from normal to either a bull’s eye maculopathy or total atrophy of the macular region.^1^ A considerable amount of individuals with COD also develop rod dysfunction, leading to a cone-rod dystrophy (CRD) with panretinal degeneration. In CRD, the loss of rod function can also be concomitant with the loss of cone function. So, apart from the loss of central vision, individuals with CRD also experience night blindness and loss of peripheral vision, leading to legal blindness at an earlier age.^1,6^

Molecular genetic studies have identified five genes mutated in individuals with ACHM, eight genes implicated in COD, and 17 genes implicated in CRD (RetNet, see Web Resources).^1,7–9^ Cone disorders can follow all modes of Mendelian inheritance and manifest as nonsyndromic and syndromic forms.^1,2^ Cone-disease-associated genes encode proteins that fulfill crucial roles in the cone phototransduction cascade, transport processes toward or through the connecting cilium, cell membrane morphogenesis and maintenance, synaptic transduction, and the retinoid cycle.^1,7–9^

Whole-exome sequencing (WES) has proven to be very effective in the discovery of genetic defects in inherited retinal diseases.^10–14^ Here, we report the identification by WES of mutations in *POC1B* (MIM 614784), encoding a protein previously associated with basal body stability,^15^ underlying autosomal-recessive COD or CRD. In addition, we provide an integrated functional approach to substantiate the causality of the identified mutations.

## Material and Methods

### Subjects and Clinical Evaluation

**Figure 1:**
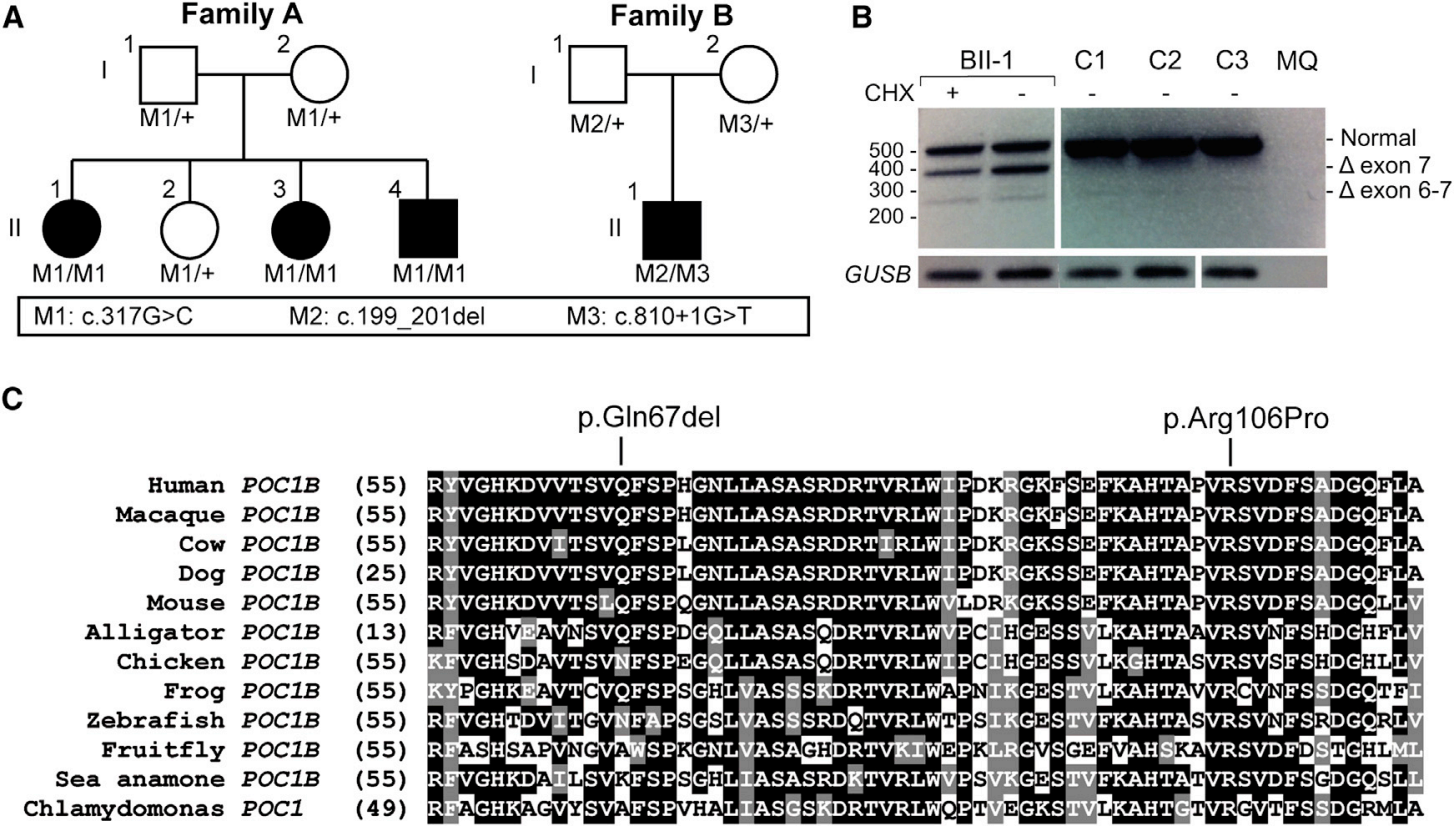
*POC1B* Mutations in Families Affected by COD or CRD (A) Sanger sequencing showed the segregation of the homozygous missense mutation M1 (c.317G>C [p.Arg106Pro]) in family A and mutations M2 (c.199_201del [p.Gln67del]) and M3 (c.810+1G>T) in family B. (B) mRNA RT-PCR studies showed a normal 421 bp product and aberrant 387 and 271 bp products lacking exon 7 and exons 6 and 7, respectively. The 387 and 271 bp cDNA deletions result in the predicted truncated POC1B products p.Val226Glyfs^∗^30 (c.677_810del) and p.Phe188Aspfs^∗^73 (c.561_810del), respectively. (C) Evolutionary conservation of amino acid residues Gln67 and Arg106 in POC1B. The glutamic acid at position 67 is moderately conserved, whereas the arginine at position 106 is completely conserved among the listed species. Identical amino acids are indicated in black boxes, and conserved residues are indicated in gray boxes.

A nonconsanguineous Turkish family (family A) with three siblings affected by COD and CRD and an isolated Dutch individual (family B) with ACHM that evolved into a progressive retinal dystrophy were included in this study (Figure 1A). These families belong to a large cohort of individuals affected by ACHM (n = 21), COD (n = 110), or CRD (n = 112). Most of the probands are the only affected persons in their family, and they were ascertained in various ophthalmic centers in the Netherlands, Belgium, the United Kingdom, and Canada. The individuals diagnosed with ACHM had a history of congenital pendular nystagmus, reduced visual acuity, photophobia, and poor or absent color vision in early infancy and had absent cone function (but normal rod responses) on ERG. Individuals in our cohort were classified as having COD when they presented with a childhood or early-adult-onset progressive deterioration of visual acuity and color vision, reduced cone responses on ERG, and normal rod responses for ≥5 years.^6^ Inclusion criteria for CRD were progressive loss of central vision, color-vision disturbances, and a reduction of both cone (equally or more severely reduced) and rod responses on ERG.^6^

After identification of the genetic defect, the medical records of the affected individuals of families A and B were evaluated. Ophthalmologic examinations were performed on several occasions and included best-corrected visual acuity (Snellen chart), slit-lamp biomicroscopy, ophthalmoscopy, color-vision testing (Hardy-Rand-Rittler color-vision test and Lanthony panel D-15 tests), and visual-field testing using Goldmann kinetic perimetry (targets V-4e and I-4e to I-1e). Fundus photographs of both the macular area and the four peripheral quadrants were available for two individuals. ERG was performed according to the protocol of the International Society for Clinical Electrophysiology of Vision.^16^ Time-domain optical coherence tomography (OCT; Stratus OCT 3000, Carl Zeiss Meditec) was obtained in one affected individual (A-II:4), and spectral-domain OCT (SD-OCT; Heidelberg Spectralis HRA+OCT, Heidelberg Engineering; 30° single-line scans, ten frames per line) was obtained in proband B-II:1. The acquisition of SD-OCT was limited as a result of nystagmus. This study was approved by the institutional review boards of the participating centers and adhered to the tenets of the Declaration of Helsinki. All subjects provided written informed consent prior to participation in the study.

### Exome Sequencing and Variant Identification

A SOLiD4 sequencing platform (Life Technologies) was utilized for WES in 12 CRD- or COD-affected probands from families with suspected autosomal-recessive inheritance, and the exomes were enriched according to the manufacturer’s protocol with the use of the SureSelect Human All Exon v.2 Kit (50 Mb), containing the exonic sequences of approximately 21,000 genes (Agilent Technologies). LifeScope software v.2.1 (Life Technologies) was used for mapping color space reads along the hg19 reference genome assembly (UCSC Genome Browser). Single-nucleotide variants were called by high-stringency calling with the DiBayes algorithm. Small insertions and deletions were detected with the SOLiD Small Indel Tool (Life Technologies). For individual A-II:1, 69,686,646 reads were uniquely mapped to the gene-coding regions; median coverage was 58.2×, and there were 44,784 sequence variants. For proband B-II:1, 75,493,298 reads were uniquely mapped to the gene-coding regions; median coverage was 66.7×, and there were 47,304 sequence variants. For validating the WES sequence variants and excluding the presence of other *POC1B* mutations, all coding exons and exon-intron boundaries of *POC1B* were amplified with primers designed with Primer 3 software (Table S1, available online).

### mRNA Analysis by RT-PCR

For assessing the effect of c.810+1G>T on the *POC1B* transcript, total RNA was isolated from peripheral-blood cells from the affected person B-II:1 and three control individuals according to the manufacturer’s (QIAGEN) protocol. The peripheral-blood cells of B-II:1 and the control individuals were cultured according to standard procedures with the use of phytohaemagglutinine. The leukocytes of the affected individual were grown for 4–6 hr with or without cycloheximide for visualization of the effect of the mutation and possible degradation of nonsense-containing mRNAs by nonsense-mediated decay.^17^ With the use of a hypotone osmotic shock and centrifugation, the leucocytes were separated from the erythrocytes. Reverse transcription with iScript (Biorad) was performed on 1 μg of total RNA. RT-PCR experiments were performed with 2.5 μl cDNA with primers in exons 5 and 9 (Table S2) (35 cycles) and were followed by Sanger sequencing using a 3100 or 3730 DNA Analyzer (Applied Biosystems).

### Zebrafish Morpholino Knockdown

Tupfel long fin zebrafish were bred and raised under standard conditions.^18^ All experiments were carried out in accordance with European guidelines on animal experiments (2010/63/EU). Zebrafish eggs were obtained from natural spawning. Antisense morpholino oligonucleotides (MOs) blocking the translation (5′-GATCCTCCATTACAGACGCCATGAT-3′) or the 5′ splice site of exon 2 (5′-AAGTTCTCTGTCTTATTCAGGAGGA-3′) of *poc1b*^15^ were obtained from GeneTools. A MO directed against a human β-globin intronic mutation (5′-CCTCTTACCTCAGTTACAATTTATA-3′) was used as a standard negative control. For MO knockdown, 1 nl of diluted MO (ranging from 2 to 10 ng) was injected into the yolk of 1- to 2-cell-stage embryos with a Pneumatic PicoPump pv280 (World Precision Instruments). A minimum sample size of 80 larvae was used in each injection experiment. After injection, embryos were raised at 28.5°C in E3 embryo medium (5 mM NaCl, 0.17 mM KCl, 0.33 mM CaCl_2_, and 0.33 mM MgSO_4_) supplemented with 0.1% (w/v) methylene blue. At 4 days postfertilization (dpf), embryos were divided into two groups on the basis of the presence of the ocular phenotype described by Pearson et al.^15^ (<15 arbitrary units on a stereomicroscope’s ocular scale bar; Zeiss). For both groups of embryos, as well as injected and uninjected controls, optokinetic responses (OKRs) were measured and histological analysis of the retina was performed.

cDNAs encoding human wild-type and variant (p.Gln67del and p.Arg106Pro) proteome of centriole 1B (POC1B, previously Pix1) were cloned in pCS2+/DEST with the use of Gateway Technology (Life Technologies), linearized, and used as templates for in vitro transcription. mRNAs were prepared with the mMESSAGE mMACHINE Kit (Life Technologies) according to the manufacturer’s instructions. A dose of 100 pg mRNA was injected with the MOs as described above.

### Zebrafish OKR Assay

The OKR was measured by a previously described method.^19^ Zebrafish larvae were mounted in an upright position in 3% methylcellulose in a small Petri dish. The Petri dish was placed on a platform surrounded by a rotating drum 8 cm in diameter. A pattern of alternating black and white vertical stripes was displayed on the drum interior (each stripe subtended an angle of 36°). Larvae (4 dpf) were visualized through a stereomicroscope positioned over the drum and illuminated with fiberoptic lights. Eye movements were recorded while larvae were optically stimulated by the rotating stripes. Larvae were subjected to a protocol of a 30 s counterclockwise rotation, a 10 s rest, and a 30 s clockwise rotation. Thereafter, the larvae were washed out of the methylcellulose and fixed for histological analysis.

### Staining of Photoreceptor Outer Segments in Zebrafish Embryos

Larvae (4 dpf) tested for OKR were fixed in 4% formaldehyde in PBS for 24 hr, dehydrated through graded ethanol steps from 70% to 100%, and embedded via a standard protocol in glycol methacrylate. The eyes were sectioned (2 μm), stained with boron-dipyrromethene (1:10,000 in PBS) for membranes and with DAPI (1:8,000) for nuclei, and imaged with a Zeiss Axio Imager Z1 fluorescence microscope.

### Immunostaining and Microscopy

Zebrafish and rat samples for immunohistochemistry (unfixed and fixed in 4% paraformaldehyde) were rinsed in 30% sucrose in PBS and directly frozen in Tissue Tek in melting isopentane. Cryosections of unfixed adult zebrafish and rat eyes (7 μm) were stained for Poc1b with anti-hsPOC1B (1:50; generously provided by Chad Pearson and Mary Pinter, University of Colorado) as described for cultured cells by Pearson et al.^15^ Sections were counterstained with GT335 (1:100; mouse monoclonal antibody against polyglutamylated tubulin, kindly provided by Carsten Janke, Centre National de la Recherche Scientifique Centre de Recherches en Biochimie Macromoleculaire). Sections of fixed zebrafish morphants were stained for green and red double cones with a mouse monoclonal antibody (zpr-1, raised against Arr3a, 1:500; ZIRC) and for rods with anti-rhodopsin (1:500; Novus Biologicals). Sections were washed with PBS, permeabilized for 20 min in 0.01% (v/v) Tween-20 in PBS, and washed again. Next, sections were blocked with 10% normal goat serum and 2% BSA in PBS, and primary antibodies were incubated overnight at 4°C in blocking buffer. After washing with PBS, secondary antibodies were incubated in blocking buffer for 45 min at room temperature. Samples were counterstained with DAPI and mounted with Prolong Gold (Life Technologies). For all sections, goat anti-mouse or goat anti-rabbit (Alexa 488 or 568, respectively, 1:500; Life Technologies) secondary antibody was used.

### cDNA Constructs

All expression constructs were created with Gateway Technology (Life Technologies) according to the manufacturer’s instructions. These constructs encoded 3×HA-POC1B wild-type and variants (p.Arg106Pro and p.Gln67del) and 3×FLAG-FAM161A for coimmunoprecipitation and encoded monomeric red fluorescent protein (mRFP)-POC1B wild-type and variants and PalmMyr-CFP-FAM161A for colocalization studies. cDNA constructs encoding the full-length POC1B of 478 amino acids (POC1B; RefSeq accession numbers NM_172240.2 [gene] and NP_758440.1 [protein]) or different fragments thereof were generated by Gateway-adapted PCR and subsequently cloned.^20^ The first fragment (POC1B-WD40) spanned amino acids 1–297 and contained the WD40 domain. The second fragment (POC1B-SR) spanned amino acids 298–426 and did not hold any known domains. The third fragment (POC1B-CC) spanned amino acids 427–478 and contained a single coiled-coil domain (Figure S1). Constructs encoding POC1B variants p.Gln67del and p.Arg106Pro were generated by site-directed mutagenesis PCR. Constructs encoding the full-length FAM161A were generated from a full-length *FAM161A* clone.^21^ The sequence of all entry clones was verified by Sanger sequencing.

### Yeast Two-Hybrid Assay

The GAL4-based yeast two-hybrid system (HybriZAP, Stratagene) was used for identifying binary protein-protein interaction partners of POC1B. The construct encoding the full-length POC1B and the three constructs encoding fragments of POC1B were fused to a DNA binding domain (GAL4-BD) and used as bait for screening a human oligo-dT-primed retinal cDNA library. The yeast strain *PJ69-4A*, which carries the *HIS3* (histidine), *ADE2* (adenine), and *LacZ* (β-galactosidase) reporter genes, was used as a host. Interactions were analyzed by assessment of reporter gene (*HIS3* and *ADE2*) activation via growth on selective media and β-galactosidase colorimetric filter lift assays (*LacZ* reporter gene). cDNA inserts of clones containing putative interaction partners were confirmed by Sanger sequencing.

### Localization in hTERT-RPE1 Cells

Human TERT-immortalized retinal pigment epithelium 1 (hTERT-RPE1) cells were cultured as previously described.^22^ Cells were seeded on coverslips, grown to 80% confluency, and subsequently serum starved for 24 hr in medium containing only 0.2% fetal calf serum for inducing cilium growth. The cells were then cotransfected with constructs encoding mRFP-POC1B (wild-type or variants) and PalmMyr-CFP-FAM161A (wild-type) with the use of Lipofectamine 2000 (Life Technologies) according to the manufacturer’s instructions. Cells were fixed in 4% paraformaldehyde for 20 min, treated with 1% Triton X-100 in PBS for 5 min, and blocked in 2% BSA in PBS for 20 min. Cells were incubated with the primary antibody GT335 (cilium and basal body marker, 1:500) and α-RPGRIP1L (SNC039 + SNC040,^23^ transition zone marker, 1:500), diluted in 2% BSA in PBS, for 1 hr. After washing in PBS, the cells were incubated with the secondary antibody for 45 min. Secondary antibodies goat anti-mouse, goat anti-guinea pig, and goat anti-rabbit (Alexa 488, 568, and 647, respectively, 1:500; Life Technologies) were diluted in 2% BSA in PBS. Cells were washed with PBS and briefly with milliQ before being mounted in Vectashield containing DAPI (Vector Laboratories). The cellular localization of wild-type and variant POC1B proteins was analyzed with a Zeiss Axio Imager Z1 fluorescence microscope equipped with a 63× objective lens. Optical sections were generated through structured illumination by the insertion of an ApoTome slider into the illumination path and subsequent processing with AxioVision (Zeiss) and Photoshop CS4 (Adobe Systems) software.

### Coimmunoprecipitation

3×HA-POC1B (wild-type and variants) and 3×FLAG-FAM161A were cosynthesized in human embryonic kidney 293T (HEK293T) cells. As a negative control, the functionally unrelated p63 was cosynthesized with both POC1B and FAM161A. As positive controls, the previously described interactions between nephrocystin-4 (encoded by *NPHP4* [MIM 607215]) and RPGRIP1^24^ and between lebercilin (encoded by *LCA5* [MIM 611408]) and FAM161A were used.^21^ After 48 hr of expression of these genes, cells were lysed on ice in lysis buffer (50 mM Tris-HCL [pH 7.5], 150 mM NaCl, and 0.5% Triton X-100) supplemented with complete protease inhibitor cocktail (Roche). Lysates were incubated with anti-HA affinity matrix (Roche) or with anti-FLAG M2 agarose from mouse (Sigma-Aldrich) for 5 hr at 4°C. After incubation, beads with bound protein complexes were washed in lysis buffer and subsequently taken up in 4× NuPAGE Sample Buffer and heated for 10 min at 70°C. Beads were precipitated by centrifugation, and supernatant was run on a NuPAGE Novex 4%–12% Bis-Tris SDS-PAGE gel. The interaction between 3×HA-POC1B and 3×FLAG-FAM161A was assessed by immunoblotting, followed by staining with either monoclonal mouse anti-HA or monoclonal mouse anti-FLAG (1:1,000; Sigma-Aldrich) as a primary antibody and goat anti-mouse IRDye800 (1:20,000; Li-Cor) as a secondary antibody. Fluorescence was analyzed on a Li-Cor Odyssey 2.1 infrared scanner.

## Results

### Identification of *POC1B* Mutations

To localize the genetic defect in a Turkish family with three siblings affected by COD or CRD (family A; Figure 1A), we performed exome sequencing in A-II:1. Putative causal mutations were selected when present with a frequency < 0.5% in dbSNP and our in-house controls (n = 2,604 alleles) and when they represented nonsense, frameshift, canonical splice-site, or missense mutations with a PhyloP score > 2.7 (range −14.1–6.4).^25^ Under the assumption of autosomal-recessive inheritance, we identified potential compound-heterozygous mutations (present in >20% sequence-variant reads) in *ASTE1*, *CNTN3* (MIM 601325), and *TUBGCP2*; Sanger sequencing of these mutations showed that they did not segregate with the disease. We identified one potential homozygous mutation (present in >80% sequence-variant reads; Table S3), c.317C>G (p.Arg106Pro) in *POC1B* (MIM 614784), and upon Sanger sequence analysis, it was found to be present in a homozygous state in the three affected siblings and in a heterozygous state in the parents and unaffected sibling (Figure 1A). The arginine at position 106 is highly conserved up to *Chlamydomonas* (Figure 1C). The c.317 position has a high PhyloP score of 6.1, and the c.317C>G mutation was not identified in 189 ethnically matched controls or in the NHLBI Exome Sequencing Project Exome Variant Server (EVS, release ESP6500).

Using the same stringent sequence-variant filtering as for family A, exome sequencing in the Dutch family B (proband B-II:1, diagnosed with atypical ACHM; Figure 1A) identified three genes with potential compound-heterozygous mutations (Table S4). The sequence variants in *NUDT14* (MIM 609219) and *PIKFYVE* (MIM 609414) did not segregate with the disease. In *POC1B*, we identified a 3 nt deletion, c.199_201del (p.Gln67del), and a mutation affecting a canonical splice-site nucleotide, c.810+1G>T. By RT-PCR of this individual’s lymphoblast mRNA, the latter mutation was shown to induce skipping of exons 6 and 7 (c.561_810del; minor mutant product) or exon 7 (c.677_810del; major mutant product) (Figure 1B) and thus result in the predicted truncated proteins p.Phe188Aspfs^∗^73 and p.Val226Glyfs^∗^30, respectively. Segregation analysis confirmed that both parents carry one of these *POC1B* mutations (Figure 1A). Neither mutation was identified in 149 ethnically matched controls or the NHLBI EVS. The glutamine at position 67 is moderately conserved up to *Xenopus* (Figure 1C).

Genome-wide SNP homozygosity-mapping data of >400 unrelated individuals with autosomal-recessive CRD, Leber congenital amaurosis, and retinitis pigmentosa (RP [MIM 268000]) were assessed through the European Retinal Disease Consortium^10,26^ and allowed the identification of eight probands with a large homozygous region spanning *POC1B*. However, Sanger sequencing of *POC1B* in these probands did not reveal additional pathogenic mutations. Subsequent Sanger sequence analysis of *POC1B* in a more specific cohort, i.e., in individuals diagnosed with ACHM (n = 21), COD (n = 110), or CRD (n = 112), also did not reveal additional individuals with *POC1B* mutations.

### Clinical Features of Affected Individuals of Families A and B

**Figure 2:**
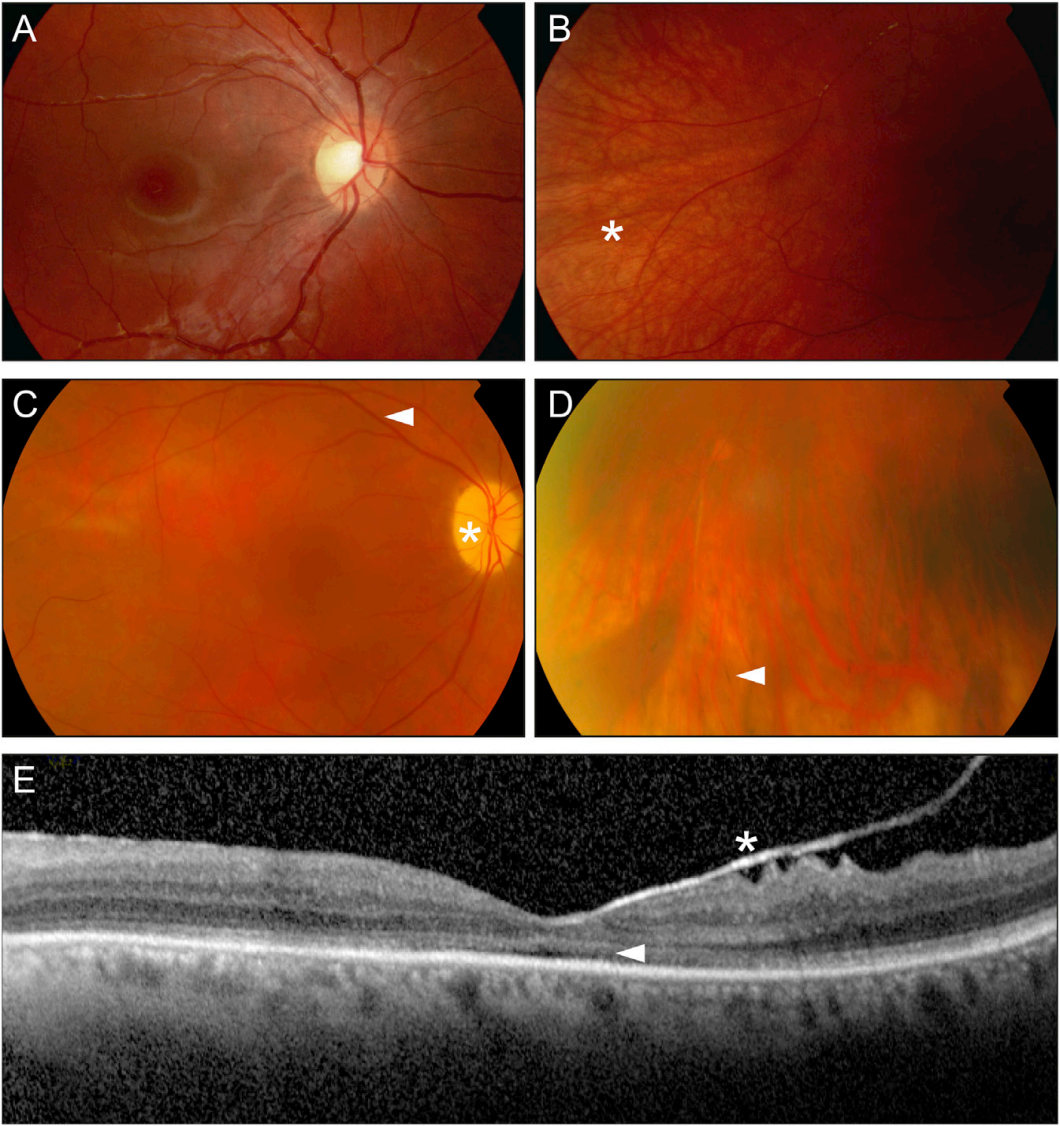
Clinical Presentation of Subjects with *POC1B* Mutations (A and B) Fundus photography of the right eye of individual A-II:3 of family A at 12 years of age. (A) Posterior pole with normal macular region. (B) Peripheral field with relative hypopigmentation (^∗^), but no pathologic RPE changes. (C and D) Fundus photography of the right eye of individual B-II:1 of family B at 60 years of age. (C) Posterior pole with optic nerve pallor (^∗^), attenuated vessels (arrowhead), and no RPE disturbances at the macula. (D) Peripheral inferior field with mild RPE atrophy and bone-spicule pigmentations (arrowhead). (E) OCT of the left eye of individual B-II:1 showed no foveal hypoplasia but did show changes at the inner-segment ellipsoid zone (arrow) and adherent posterior hyaloid membrane (^∗^).

Table 1 presents a summary of the clinical features of the four individuals with *POC1B* mutations. Fundus and OCT images are depicted in Figure 2. The proband (A-II:1) of family A presented with reduced visual acuity in early infancy and mild nystagmus. The diagnosis of incomplete ACHM was contemplated on the basis of ERG showing absent cone function but normal rod responses at the age of 9 years. In the following years, however, visual acuity seemed to deteriorate, and a few years later, her two younger siblings experienced a rapid loss of central vision, suggesting COD (Figures 2A and 2B) in two siblings and CRD in one sibling. No long-term data are available on these three persons. In contrast, B-II:1 was followed for more than 40 years. He was also diagnosed with ACHM in childhood on the basis of the classical signs of reduced visual acuity, photophobia, nystagmus, very poor color vision, and matching ERG responses. In his fifth decade, visual acuity began to drop slowly, and in his sixth decade, degenerative changes consisting of RPE atrophy and bone-spicule pigmentations were noted in the periphery of the inferior quadrant (Figures 2C and 2D). On OCT, changes at the inner-segment ellipsoid zone were observed, suggesting the loss of junctions between inner and outer segments (Figure 2E). ERG at 55 years of age showed absent cone and significantly reduced rod responses. Altogether, the diagnosis changed from isolated cone dysfunction to progressive cone-rod disease. Systematically, he was treated for hypertension and had normal renal function.

### Localization of POC1B in Human hTERT-RPE1 Cells

**Figure 3:**
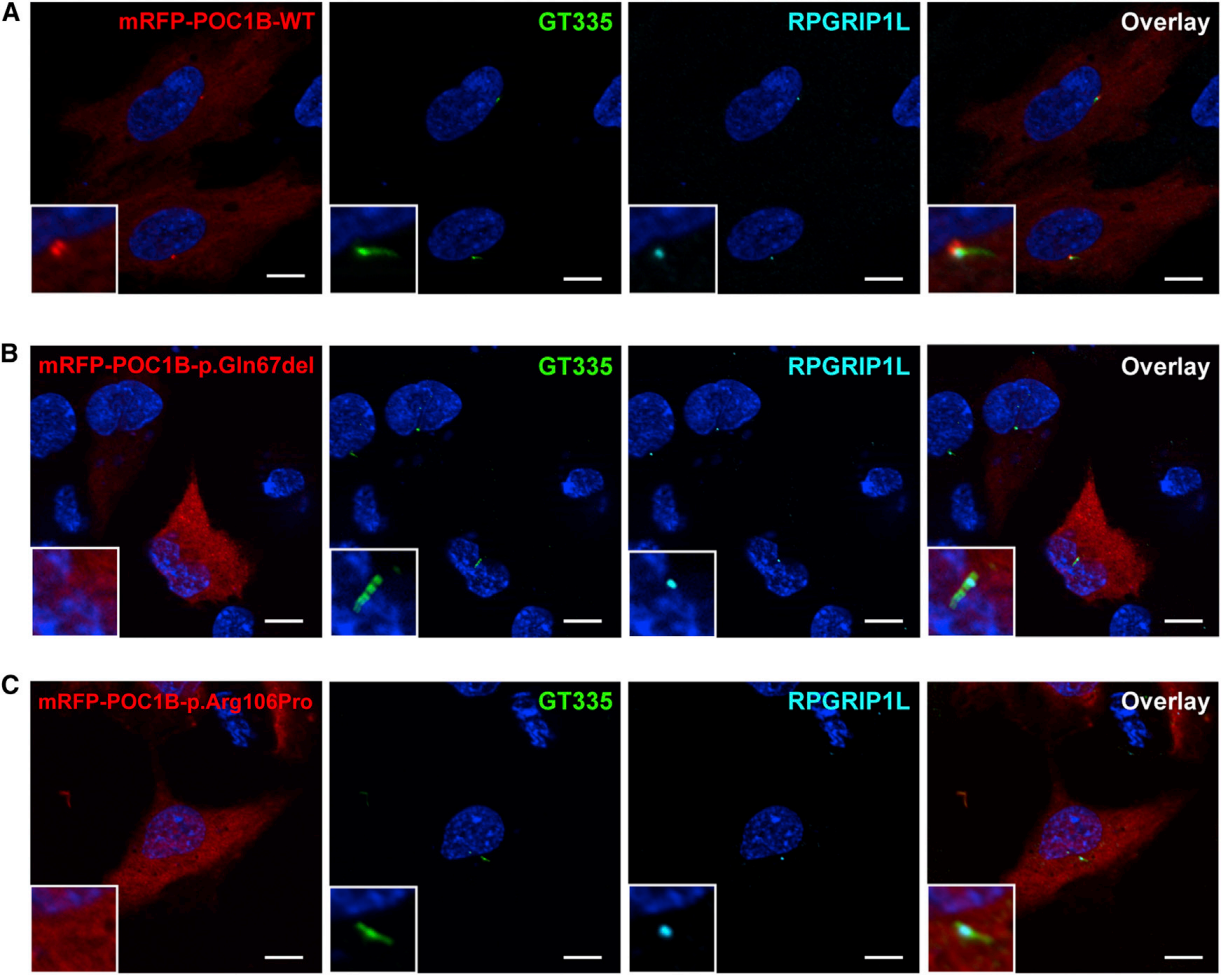
Subcellular Localization of Wild-Type and Variant POC1B in hTERT-RPE1 Cells Localizations of wild-type mRFP-POC1B (A), mRFP-POC1B-p.Gln67del (B), and mRFP-POC1B-p.Arg106Pro (C) (all in red). Additional images are shown in Figure S2. Cilia were counterstained with the basal body and cilium marker GT335 (green) and transition-zone marker RPGRIP1L (cyan). Wild-type mRFP-POC1B showed cytosolic localization with enrichment at the basal body region, as seen in the magnifications in the insets. Both variants showed similar cytosolic localization but lacked enrichment at the base of the cilium. In all pictures, nuclei were stained with DAPI (blue). Scale bars represent 10 μm.

To investigate the effect of the identified *POC1B* mutations on the subcellular localization of the encoded protein, we synthesized wild-type and variant recombinant POC1B proteins, fused to mRFP, in ciliated hTERT-RPE1 cells. Wild-type POC1B localization was concentrated at the ciliary basal body, as indicated by costaining with the anti-polyglutamylated tubulin antibody GT335, although some diffuse cytoplasmic localization was also observed (Figure 3A; Figure S2A). This confirms previous results of Venoux et al.^20^ In contrast, variant POC1B proteins, carrying either the p.Gln67del (Figure 3B; Figure S2B) or the p.Arg106Pro (Figure 3C; Figure S2C) amino acid change, completely lost their ciliary localization.

### Localization of Poc1b in Photoreceptor Cells

**Figure 4:**
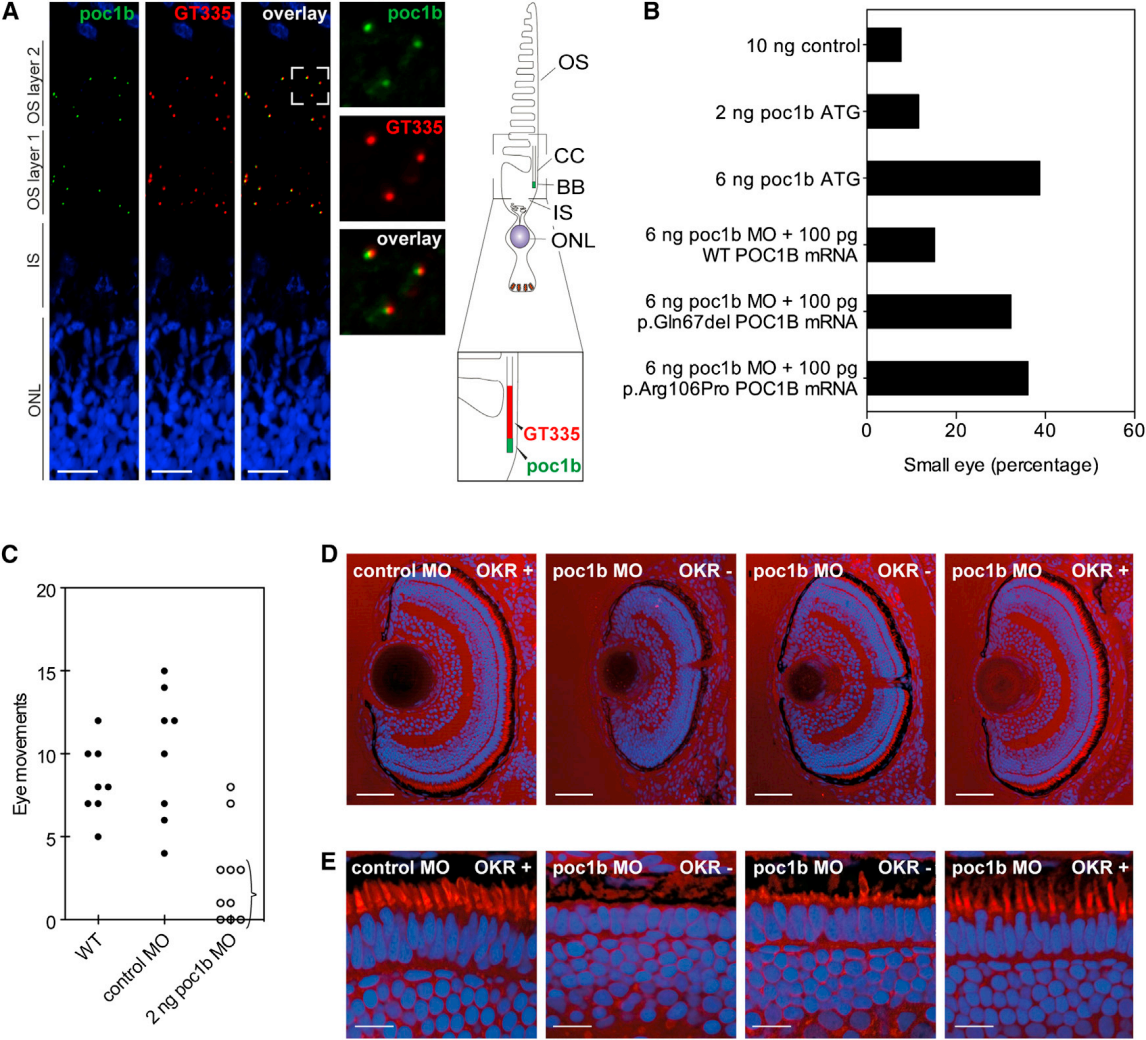
Morphological and Functional Effects of MO Knockdown of *poc1b* in Zebrafish Larvae (A) Localization of Poc1b (green) in the retina of adult zebrafish. The retina of adult zebrafish typically contains two layers of photoreceptor outer segments and associated basal bodies (OS layers 1 and 2). Both layers showed Poc1b immunoreactivity overlapping with and adjacent to staining of GT335 (red), a marker of the connecting cilium. Abbreviations are as follows: OS, outer segment; CC, connecting cilium; BB, basal body; IS, inner segment; and ONL, outer nuclear layer. The scale bar represents 15 μm. (B) Phenotypic analysis of morphant eyes. Injection of 6 ng translation-blocking MO led to a higher number of small eyes than in control-MO-injected larvae. The phenotype could be partially rescued by coinjection of wild-type *POC1B* mRNA, but not c.199_201del (p.Gln67del) or c.317C>G (p.Arg106Pro) mutant *POC1B* mRNA. (C) Analysis of the OKR (Movies S1 and S2). Larvae with decreased responses were already observed in a pool of larvae injected with 2 ng MO (indicated with an accolade). At this dose, there were also morphants that did respond to the visual stimulus. (D) Sections of eyes injected with control or *poc1b* MOs were stained for outer segments (red) and nuclei (blue). Eyes of morphants that did not respond to the visual stimulus were smaller. The scale bar represents 50 μm. (E) Outer segments were decreased in length or absent in nonresponsive morphants (OKR^−^), whereas outer segments were normal in length in responsive larvae (OKR^+^). The scale bar represents 10 μm.

The retinal function of Poc1b was evaluated in zebrafish. Staining with anti-human POC1B showed that the protein was located at the basal bodies of both the inner and the outer photoreceptor layers of the adult zebrafish retina (Figure 4A). In a focal plane different from the basal bodies, some staining was also observed at the outer limit of the outer nuclear layer (data not shown). Poc1b immunostaining was also detected at the basal body of rat photoreceptors cells (Figure S3).

### *Poc1b* Knockdown Results in Visual Impairment in Zebrafish

*Poc1b* morphants display the typical ciliopathy phenotypes previously described by Pearson et al.,^15^ including pericardial edema, small eyes, pigment mislocalization, and a shortened and curved body axis (Figure S4A). Small eyes were already observed in larvae treated with 2 ng MO and became more frequent as the dose increased (Figure 4B; Figure S4B). At a dose of 6 ng MO, smaller eyes occurred in 39.8% of morphants and were on average significantly smaller than wild-type eyes (Figure 4B; Figure S4B), and 92.5% of morphants displayed one or more of the phenotypes described above without higher mortality than in controls. Specificity of the MO knockdown was previously verified with a second MO targeting the 5′ splice site of *poc1b* exon 2, which resulted in 59.1% of morphants with small eyes (Figure S4B).

The OKR was assessed in larvae injected with control and *poc1b* MOs. OKR was absent or lower in morphants with small eyes than in wild-type or control-MO-injected larvae (Figure 4C; Movies S1 and S2). Morphants that received the same dose of MO but had normal-sized eyes responded normally to the OKR stimulus. This phenotype was confirmed in larvae treated with a splice-site-blocking MO (data not shown). Outer-segment length was not affected in control-MO-injected larvae and morphants that did show an OKR. Histological analysis of the retina of the morphants subjected to OKR measurement revealed shortened or absent outer segments of the photoreceptors, whereas lamination appeared normal (Figures 4D and 4E). We observed a perfect correlation between the small-eye phenotype and a diminished or absent OKR. The size of the eyes appeared to correlate with outer-segment length and responsiveness to visual stimuli. As such, we could quantify the size of the eye to measure the effects of loss of Poc1b function. Indeed, coinjection of 100 pg human wild-type *POC1B* mRNA, but not of c.199_201del (p.Gln67del) or c.317C>G (p.Arg106Pro) mutant *POC1B* mRNA, significantly rescued *poc1b* knockdown (Figure 4B; Figure S4B).

Immunohistochemical staining for typical rod (rhodopsin) and cone (zpr-1) markers was absent from a subset of cells in morphants with smaller eyes (Figure S4C). High-magnification pictures showed that whereas immunostaining was absent in certain regions of the morphant retina, the nuclei of the photoreceptor cells were still present.

### Identification of a Retinal Protein Interacting with POC1B

**Figure 5:**
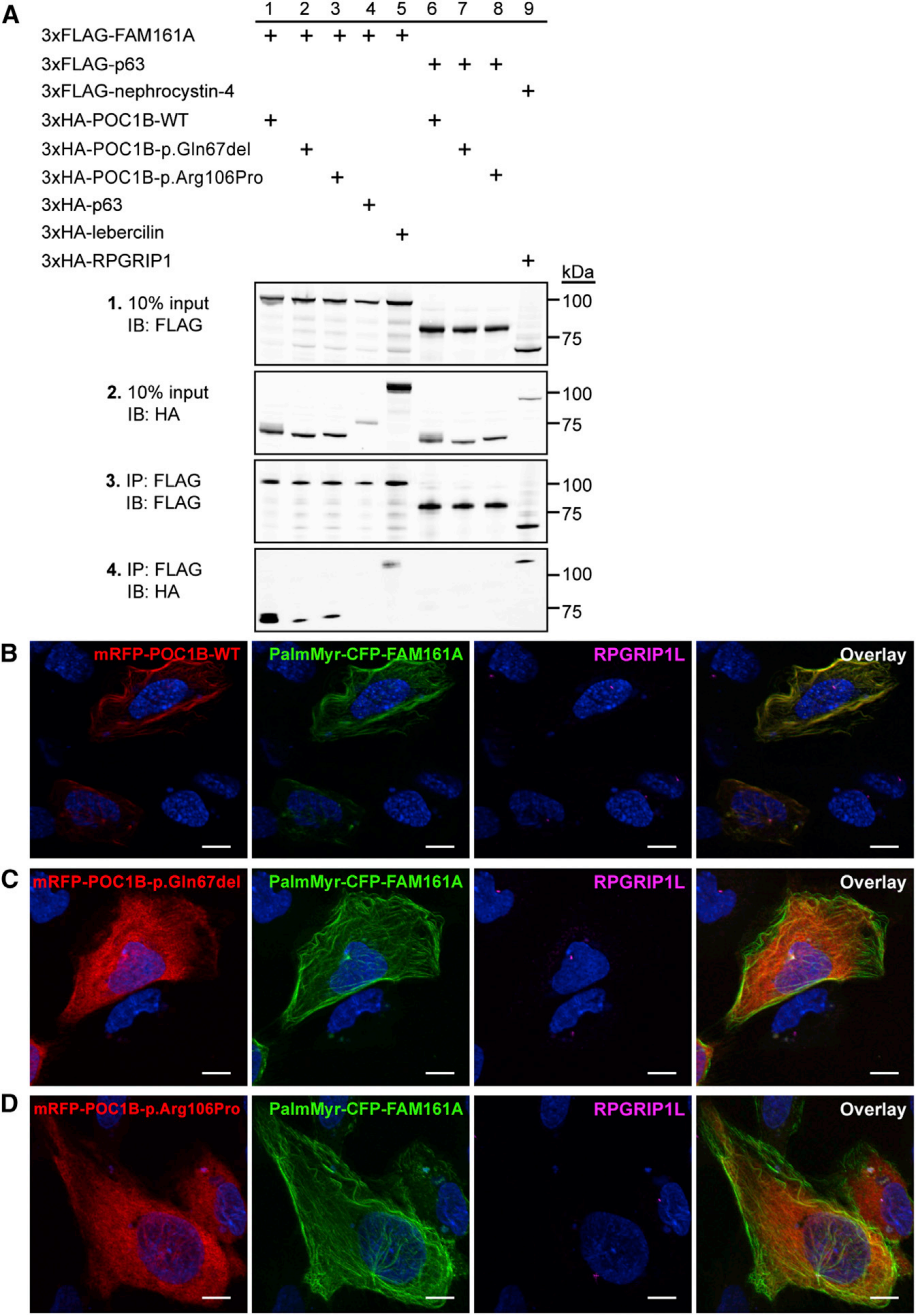
Coimmunoprecipitation and hTERT-RPE1 Localization Studies of POC1B and FAM161A (A) Coimmunoprecipitation assay in HEK293T cells. Wild-type 3×HA-POC1B efficiently coprecipitated with 3×FLAG-FAM161A (lane 1), but coprecipitation was reduced for variants 3×HA-POC1B-p.Gln67del and 3×HA-POC1B-p.Arg106Pro (panel 4, lanes 2 and 3). Specificity was confirmed by inclusion of the unrelated p63, which failed to coimmunoprecipitate with wild-type and variant POC1B. As positive controls, coimmunoprecipitation of lebercilin (encoded by *LCA5*) by FAM161A and of RPGRIP1 by nephrocystin-4 (encoded by *NPHP4*) was used. Immunoblots of the input are shown in panels 1 and 2, and immunoblots of the FLAG immunoprecipitates are shown in panels 3 and 4. Size markers are depicted in kDa. (B) Colocalization in hTERT-RPE1 cells. PalmMyr-CFP-FAM161A (green) was targeted to the cell membrane and microtubules and translocated wild-type mRFP-POC1B (red) from the cytosol toward the cell membrane and microtubules. (C and D) This translocation by PalmMyr-CFP-FAM161A (green) was not observed for mRFP-POC1B-p.Gln67del (C, red) or mRFP-POC1B-p.Arg106Pro (D, red), which both maintained their cytosolic localization. RPGRIP1L (magenta) was used as a transition-zone marker of the cilium. Nuclei were stained with DAPI (blue). Scale bars represent 10 μm. Additional images are shown in Figure S7.

To identify interaction partners of POC1B in the retina, we employed a GAL4-based interaction trap screen in yeast (yeast two-hybrid system). We screened a library expressing human retinal cDNAs for potential interactors of POC1B. Both a construct expressing full-length POC1B and constructs expressing different POC1B fragments were used as baits (Figure S1). The fragment containing the carboxy-terminal coiled-coil domain of POC1B was found to putatively interact with five different proteins, including FAM161A (Figure S5A). The interaction with this known retinal-disease-associated protein^21,27,28^ caught our attention and was confirmed by a coimmunoprecipitation assay using both full-length proteins (Figure 5A). In the same assay, we investigated the effect of the identified missense and single-amino-acid deletion variants in POC1B on the interaction. Indeed, a significantly lower amount of altered POC1B than wild-type protein coprecipitated with FAM161A, indicating a disrupted physical interaction. The unrelated p63 did not coprecipitate with either POC1B or FAM161A, which confirmed specificity of the interaction between POC1B and FAM161A in the coimmunoprecipitation assay. The interaction between wild-type POC1B and FAM161A, and the decreased interaction between variant POC1B proteins and FAM161A, was confirmed by reciprocal coimmunoprecipitation (Figure S5B). Uncropped images of the immunoblots are shown in Figure S6.

To validate the loss of interaction between FAM161A and variant POC1B in mammalian cells, we cotransfected hTERT-RPE1 cells with constructs encoding wild-type and variant mRFP-POC1B together with PalmMyr-CFP-FAM161A (Figures 5B–5D; Figures S7A–S7C). The PalmMyr tag provides residues for palmitoylation and myristoylation, which both induce membrane association of the protein of interest.^29^ This can subsequently be visualized by fluorescence microscopy of the expression of the fluorescent CFP tag. Coexpression of *FAM161A* and *POC1B* showed complete colocalization of the encoded proteins at the plasma membrane, basal body, and association with the microtubule network. When either one of the mutations was present in the *POC1B* construct, the colocalization with FAM161A was lost completely. PalmMyr-CFP-FAM161A then maintained its membrane, basal body, and microtubule association, but the localization of variant POC1B was cytosolic without enrichment at specific subcellular sites (Figures 5C and 5D; Figures S7B and S7C).

## Discussion

In this study, we identified three *POC1B* mutations that cause autosomal-recessive COD or CRD. In two siblings of one family, loss of central vision was observed in childhood, consistent with progressive cone disease; however, in another sibling and in one isolated individual, poor visual acuity and nystagmus were present from early infancy, suggesting a form of ACHM. In the latter two individuals, visual acuity also deteriorated over time, and in one of them, peripheral retinal degeneration was observed in the sixth decade. Although there is overlap in genes associated with either ACHM or COD as a result of mutations in *CNGA3* (MIM 600053) and *CNGB3* (MIM 605080),^5,30,31^ there are, to our knowledge, no reports on the natural history of ACHM concerning peripheral degeneration.

POC1B is one of the two POC1 homologs that function together as a highly conserved core centriole and basal body component in vertebrates,^15,32–34^ invertebrates,^35,36^ and even *Chlamydomonas reinhardtii*^32^ and *Tetrahymena thermophila*.^33^ The other POC1 homolog, encoded by *POC1A* (previously *Pix2* [MIM 614783]), shows protein structure and intracellular localization similar to those of POC1B.^20,36^ Studies in *Tetrahymena thermophila* suggest that POC1 proteins are essential for both structure and stability of the basal body.^15^ Depletion studies show that POC1B, unlike POC1A, is necessary for ciliogenesis, and typical ciliopathy-associated developmental defects (e.g., curved body axis, kidney cysts, and laterality defects) were described in *poc1b* morphant zebrafish. Interestingly, they were also reported to exhibit smaller eyes, but a more detailed ophthalmological analysis was not undertaken.^15^

In light of the retinal phenotype we observed in affected individuals with *POC1B* mutations and the reported smaller eyes in *poc1b* morphant zebrafish, we disrupted *poc1b* expression by using the same translation-blocking MO used by Pearson et al.^15^ An accurate evaluation of Poc1b function in the eyes indeed confirmed that the protein is required for normal vision, given that the Poc1b-depleted zebrafish showed a severely decreased OKR in combination with smaller eyes (Figure 4C). Analysis of morphant eyes revealed decreased length of photoreceptor outer segments in the cone-dominated larval retina (Figures 4D and 4E). Poc1b appeared to be present at all basal bodies of vertebrate photoreceptors, suggesting that loss of function affects both rods and cones (Figure 4A; Figure S3). Indeed, knockdown of *poc1b* reduced immunoreactivity for important proteins in the light-transduction cascade of rod and cones alike (Figure S4C). This corresponds with the decreased visual response of *poc1b* morphants, measured in the OKR assay. Rescue of the smaller eyes associated with this phenotype was achieved with wild-type human *POC1B* mRNA.

The affected amino acids identified in this study are moderately or highly conserved in evolution (Figure 1C), and both affect the N-terminal WD40 domain (Figure S1). The third mutation alters the splice site of exon 7 and results in a truncation of the protein within the last WD40 repeat. This WD40 domain, but not the C-terminal region of POC1, has been demonstrated to be sufficient for targeting POC1 localization to centrioles.^32^ Indeed, whereas wild-type POC1B localized to the basal bodies, as previously reported, both p.Gln67del and p.Arg106Pro variant POC1B revealed a loss of association with the basal body of the cilium (Figure 3). The effect of the variants on Poc1b in zebrafish was addressed by coinjection of a *poc1b* MO in combination with human *POC1B* mRNA carrying either one of the mutations. In contrast with coinjection of wild-type mRNA, coinjection of the mutated mRNA could not induce (partial) rescue of the ocular phenotype, confirming the disturbing retinal effect of the variant amino acid residues (Figure 4B).

To provide further insights into the retinal function of POC1B, we aimed to identify retinal proteins interacting with POC1B by using a GAL4-based interaction trap screen in yeast of a retinal cDNA library. Out of the four different bait fragments of POC1B employed, only the coiled-coil region was found to yield one significant interactor: FAM161A. Interestingly, mutations in *FAM161A* lead to another retinal ciliopathy, autosomal-recessive RP (RP28).^27,28^ Binding of FAM161A was validated with coimmunoprecipitation and colocalization studies (Figure 5). Although the interaction was initially detected with a fragment containing the coiled-coil region of POC1B, introduction of the two POC1B variants in the WD40 domain of the full-length protein strongly decreased its interaction with FAM161A, reiterating the structural importance of this domain. Because FAM161A was found to be a retinal-ciliopathy-associated protein,^21,37^ the decreased interaction we observed with this retina-specific protein might induce degeneration of rod photoreceptors as a result of *POC1B* mutations in individuals with CRD.

Pearson et al. showed that in the absence of Poc1b, zebrafish present with various phenotypes that point toward a syndromic ciliopathy.^15^ In contrast, the *POC1B* mutations identified in this study are associated with a much milder, nonsyndromic cone-disease phenotype in two families. Although species-specific differences might contribute to the observed phenotypic heterogeneity, on the basis of the type and combinations of mutations identified and the reduced, but not absent, interaction between the altered POC1B and the retina-specific FAM161A, it is plausible to conclude that individuals with COD have residual POC1B activity. Combinations of more severe and/or loss-of-function *POC1B* mutations therefore might be associated with syndromic forms of retinal ciliopathies, in line with the wide disease spectrum previously observed for another ciliopathy-associated gene, *CEP290* (MIM 160142).^38–41^

In conclusion, WES led to the identification of *POC1B* mutations in two unrelated families affected by autosomal-recessive nonsyndromic COD or CRD. These variants were found to disrupt the ciliary basal body localization of POC1B and its interaction with a retina-specific, RP-associated protein, FAM161A. Given that loss of Poc1b in zebrafish furthermore resulted in early-onset retinal dysfunction, this study highlights a basal body protein photoreceptor module that contains POC1B and FAM161A and is required for photoreceptor homeostasis.

## Consortia

The members of the POC1B Study Group are Karsten Boldt, Elfride de Baere, Caroline C.W. Klaver, Frauke Coppieters, David A. Koolen, Dorien Lugtenberg, Kornelia Neveling, Jeroen van Reeuwijk, Marius Ueffing, Sylvia E.C. van Beersum, and Marijke N. Zonneveld-Vrieling.

## Figures and Tables

**Table 1:** Summary of the Clinical Data of Four Individuals with *POC1B* Mutations

	**Family A**			**Family B**
	**Subject A-II:1**	**Subject A-II:3**	**Subject A-II:4**	**Subject B-II:1**
Gender	female	female	male	male
Nystagmus	present	absent	absent	present
First documented visual acuity (age)	RE: 0.16 LE: 0.16 (3 years)	RE: 0.8 LE: 0.8 (10 years)	RE: 1.0 LE: 0.8 (3 years)	RE: 0.2 LE: 0.2 (14 years)
Last documented visual acuity (age)	RE: CF LE: 0.1 (16 years)	RE: 0.3 LE: 0.2 (19 years)	RE: 0.16 LE: 0.2 (9 years)	RE: 0.05 LE: 0.05 (60 years)
Refraction, D SE (age)	RE: −6 D LE: −6 D (16 years)	RE: −1 D LE: −1 D (19 years)	RE: +0.5 D LE: plano (9 years)	RE: −2.50 D LE: −2.25 D^a^ (59 years)
Funduscopy (age)	subtle RPE disturbances in the periphery; otherwise normal (9 years)	relative hypopigmentation in the periphery; otherwise normal (12 years)	bone-spicule pigmentations in the periphery; otherwise normal (6 years)	pallor optic disc, attenuated vessels, no RPE disturbances at the macula, RPE atrophy with bone-spicule pigmentations in the periphery of the inferior quadrants (60 years)
OCT (age)	NP	NP	intact inner-segment ellipsoid zone, no foveal hypoplasia (7 years)	changes at inner-segment ellipsoid zone, no foveal hypoplasia (60 years)
Color vision (age)	tritan defect (9 years)	tritan defect in RE, anomaloscope showed red shift in both eyes (11 years)	NP	defects in all axes (55 years)
Goldmann visual field (age)	relative central scotoma, mild peripheral constriction (13 years)	relative central scotoma, mild peripheral constriction (11 years)	relative central scotoma, mild peripheral constriction (6 years)	central scotoma, peripheral constriction (58 years)
ERG (age)	nonrecordable cone responses, normal rod responses (9 and 13 years)	severely reduced cone responses, normal rod responses (11 years)	nonrecordable cone responses, severely reduced rod responses (6 years)	nonrecordable cone responses, normal rod responses (14 years); nonrecordable cone responses, significantly reduced rod responses (55 years)
Final diagnosis	COD	COD	CRD	CRD

Visual acuity is in Snellen decimals. Abbreviations are as follows: CF, counting finger; COD, cone dystrophy; CRD, cone-rod dystrophy; D, diopter; ERG, electroretinography; LE, left eye; NP, not performed; OCT, optical coherence tomography; RE, right eye; RPE, retinal pigment epithelium; and SE, spherical equivalent.

a Refractive error before cataract extraction at 59 years of age.

## Supplemental Data

Document S1. Figures S1–S7 and Tables S1–S4

Movie S1. Representative OKR Movie of a 4 dpf Morphant Injected with Control MO The striped drum was rotating at 0–30 s and 40–70 s.

Movie S2. Representative OKR Movie of a 4 dpf Morphant Injected with 2 ng *poc1b* MO The striped drum was rotating at 0–30 s and 40–70 s. Although eye movement was strongly decreased, drum rotation was observed by its reflection in the air bubbles. The larva’s heartbeat was also observed.

Document S2. Article plus Supplemental Data

## Web Resources

The URLs for data presented herein are as follows:

NHLBI Exome Sequencing Project (ESP) Exome Variant Server (EVS), http://evs.gs.washington.edu/EVS/
Online Mendelian Inheritance in Man (OMIM), http://www.omim.org/
Primer 3, http://frodo.wi.mit.edu/
Retinal Information Network (RetNet), https://sph.uth.edu/retnet/
