# Supplementary material for: Disruption of the Basal Body Protein POC1B Results in Autosomal-Recessive Cone-Rod Dystrophy
Source: Am J Hum Genet. 2014 Aug 7;95(2):131–42. doi: 10.1016/j.ajhg.2014.06.012 (PMC4129401; doi:10.1016/j.ajhg.2014.06.012)
Supplement: Document S1. Figures S1–S7 and Tables S1–S4 [file mmc1.pdf]

The American Journal of Human Genetics, Volume *95*

Supplemental Data

Disruption of the Basal Body Protein POC1B Results in Autosomal-Recessive Cone-Rod Dystrophy

Susanne Roosing, Ideke J.C. Lamers, Erik de Vrieze, L. Ingeborgh van den Born, Stanley Lambertus, Heleen H. Arts, Consortium, Theo A. Peters, Carel B. Hoyng, Hannie Kremer, Lisette Hetterschijt, Stef J.F. Letteboer, Erwin van Wijk, Ronald Roepman, Anneke I. den Hollander, and Frans P.M. Cremers

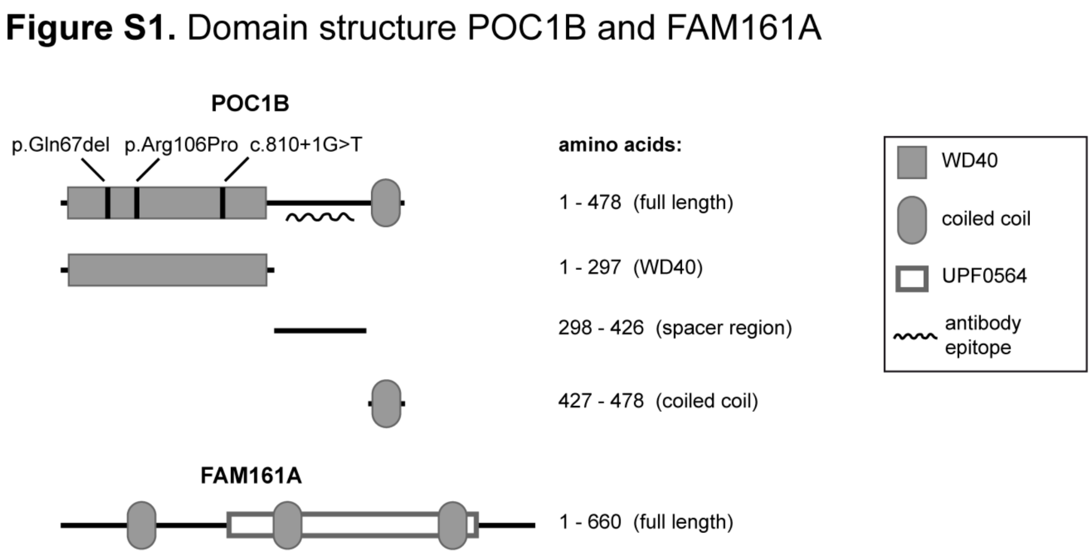

Figure S1. Domain structure POC1B and FAM161A

The *POC1B* gene encodes a 478 amino acid (aa)/ 54 kDa protein (UniProt ID: POC1B_HUMAN, Q8TC44) and is predicted to contain an N-terminal WD40 domain with seven WD40 repeats, and a C-terminal coiled-coil domain. All three variants lie within the WD40 domain. For the yeast two-hybrid screen of the retinal cDNA library, three bait fragments were generated containing either the WD40 domain, spacer region or coiled-coil domain. 0.97 x 10^6, 2.25 x 10^6, and 1.56 x 10^6 cDNA clones were screened for binary interactions, respectively. Only the coiled-coil domain yielded in-frame positive clones not previously identified as background. Two overlapping clones of *FAM161A* were identified, encoding aa 50-660 and aa 65-660 of FAM161A. Four other in-frame clones were identified, but as these did not encode ciliary nor retinal degeneration-associated proteins, none of these were further validated. The POC1B antibody was raised against the spacer region. FAM161A is a 660 aa/ 77 kDa protein (UniProt ID: F161A_HUMAN, Q3B820) that is predicted to contain three coiled-coil domains and an uncharacterized protein family UPF0564 domain.

Figure S2. Localization of wild-type and mutant POC1B in hTERT-RPE1 cells

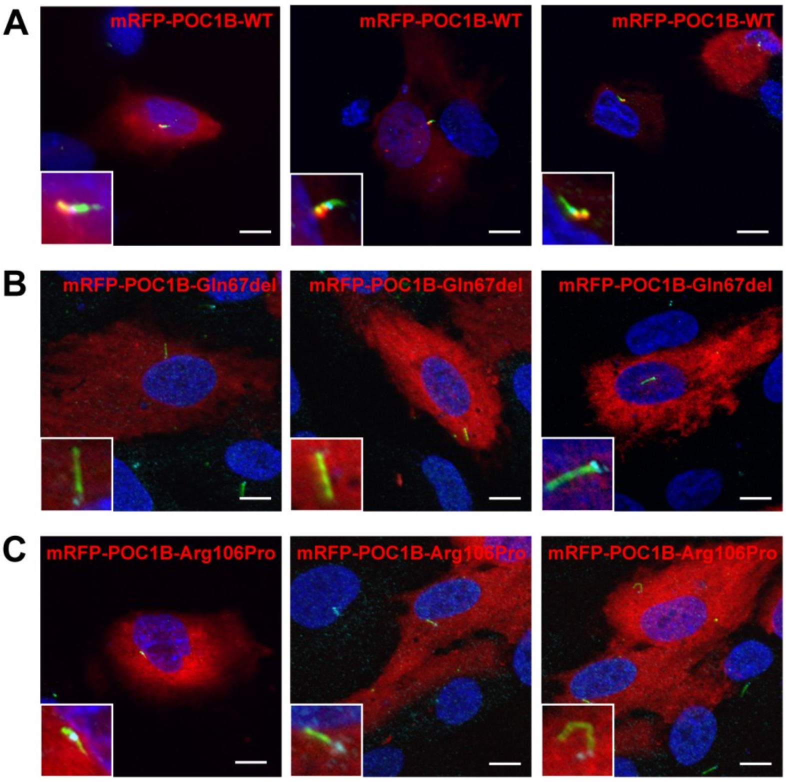

Figure S2. Localization of wild-type and variant POC1B in hTERT-RPE1 cells
Overlay photomicrographs of additional fields of hTERT-RPE1 cells corresponding to the images depicted in Figures 3A-C. (A) Overlay images of the localization of wild-type mRFP-POC1B. Cilia are counterstained with the basal-body and cilium marker GT335 (green) and transition zone marker RPGRIP1L (cyan). (B) Overlay images of the localization of mRFP-POC1B-Gln67del, and (C) mRFP-POC1B-Arg106Pro (both in red). Here, cilia are counterstained with a rabbit polyclonal antibody against ARL13B in green (1:500; Proteintech, Chicago, IL, USA) and centrosomes are counterstained with a mouse monoclonal antibody against centrin in cyan (1:500; Millipore, Billerica, MA, USA). Wild-type mRFP-POC1B shows cytosolic localization with enrichment at the basal body region, as seen in the magnifications in the insets. Both variants show similar cytosolic localization but lack the enrichment at the base of the cilium. In all pictures nuclei are stained with DAPI (blue). Scale bars: 10 µm.

Figure S3. Immunohistochemistry of Poc1b in rat retina

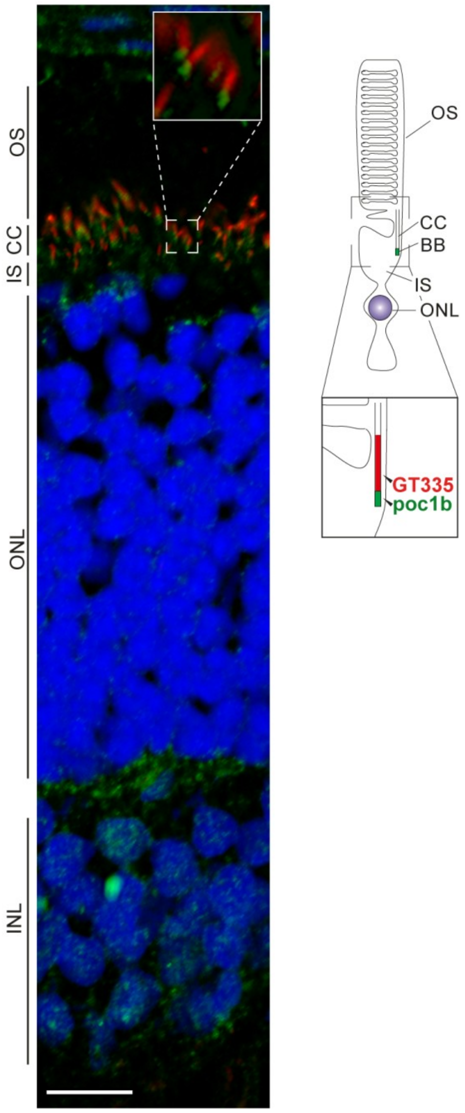

Figure S3. Immunohistochemistry of Poc1b in rat retina

Immunohistochemistry of Poc1b on a cryosection of an unfixed rat retina. Poc1b immunoreactivity in the photoreceptor is observed overlapping with, and adjacent to, GT335 staining (red), a marker of the connecting cilium. OS = outer segment, CC = connecting cilium, BB = basal body, IS = inner segment, ONL = outer nuclear layer. Scale bar = 10 µm.

Figure S4. Phenotypic analysis of *poc1b* morphant zebrafish

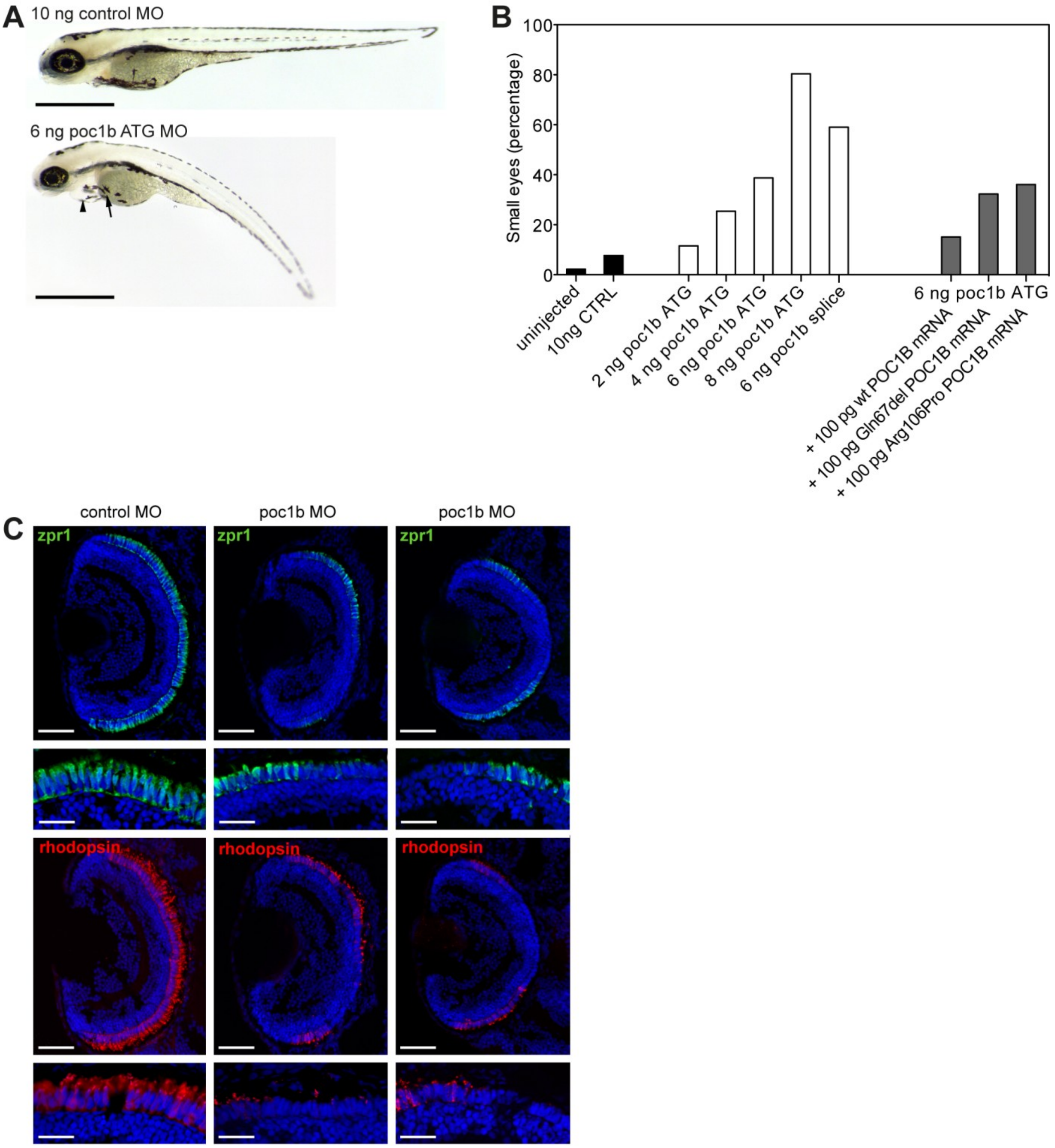

Figure S4. Phenotypic analysis of *poc1b* morphant zebrafish

(A) Morphology of *poc1b* injected larvae. Morphants had smaller eyes, curved body axis, pigment mislocalization (arrow) and pericardial edema (arrowhead). None of these phenotypes were observed in control injected animals. (B) Overview of the occurrence of small eyes in *poc1b* morpholino oligonucleotide (MO) knockdown experiments. This phenotype increased dose-dependently with the amount of injected MO. At 8 ng almost all morphants had smaller eyes, but mortality was also increased compared to controls. In all other groups mortality was similar compared to control injected animals. (C) Immunohistochemistry of cone (zpr1, green) and rod (rhodopsin, red) markers in control and morphant (6 ng) eyes. Not only were the eyes smaller, both zpr1 and rhodopsin immunoreactivity was absent in some parts of the morphant retina. Scale bar = 50 µm in low magnification pictures and 15 µm in high magnification pictures.

Figure S5. Yeast two-hybrid and coimmunoprecipitation of POC1B and FAM161A

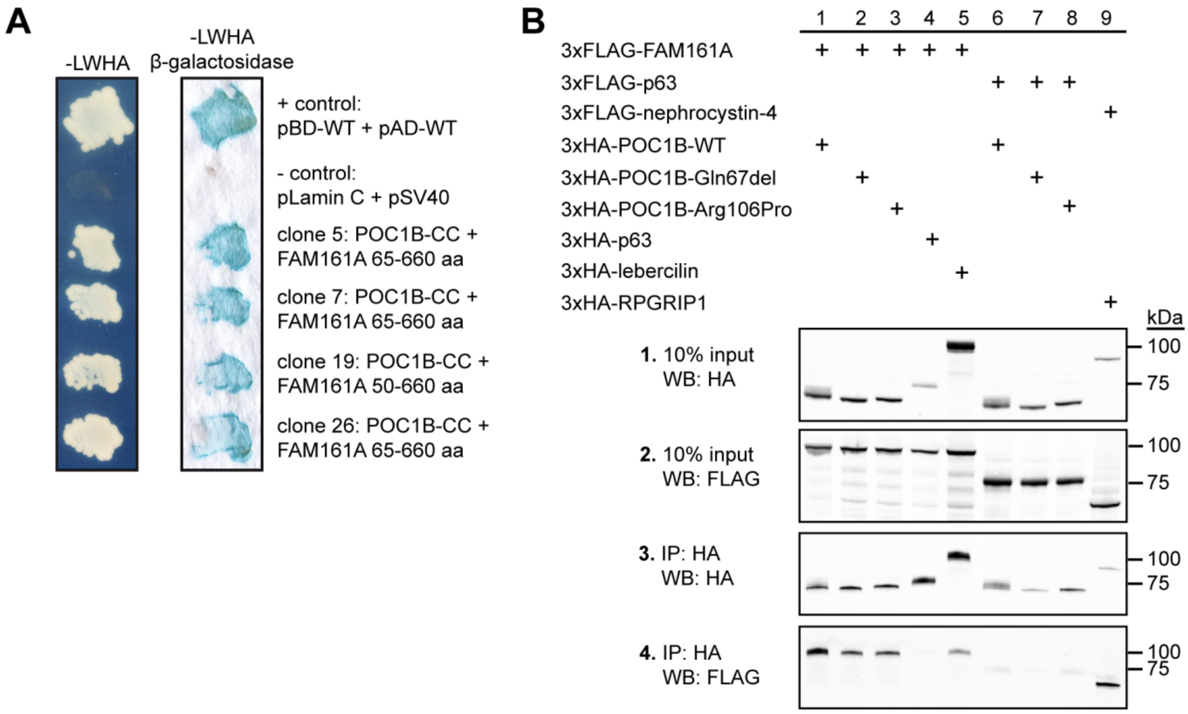

Figure S5. Yeast two-hybrid and coimmunoprecipitation of POC1B and FAM161A

(A) POC1B interacts with FAM161A in a yeast two-hybrid assay. Interactions were analyzed by assessment of reporter gene activation via growth on selective media (-LWHA) and by a colorimetric filter lift assay (-LWHA; β-galactosidase). The POC1B fragment containing the coiled-coil domain spanning amino acids (aa) 427 – 478 (POC1B-CC) was found to bind the FAM161A fragment containing aa 50-660 in one yeast clone (clone 19) and a fragment containing aa 65-660 of FAM161A in three yeast colonies (clones 5, 7, and 26). (B) Coimmunoprecipitation assay in HEK293T cells. Wild-type 3xFlag-FAM161A efficiently coprecipitated with 3xHA-POC1B using anti-HA antibodies (panel 4, lane 1). Introduction of the POC1B variants p.Gln67del and p.Arg106Pro reduced the coimmunoprecipitation efficiency (panel 4, lanes 2 and 3). Specificity was confirmed by including the unrelated 3xFLAG-p63 protein, which failed to coprecipitate significantly with wild-type and mutant 3xHA-POC1B, and the 3xHA-p63 protein, which did not coprecipitate with 3xFLAG-FAM161A. As positive controls of the coimmunoprecipitation assay, coprecipitation of 3xHA-lebercilin with 3xFLAG-FAM161A, and of 3xHA-RPGRIP1 with 3xFLAG-nephrocystin-4 were used. Immunoblots of 10% of the input are shown in panels 1 and 2, immunoblots of the HA immunoprecipitates in panels 3 and 4. Size markers are depicted in kDa.

Figure S6. Western blots of coimmunoprecipitation of POC1B with FAM161A

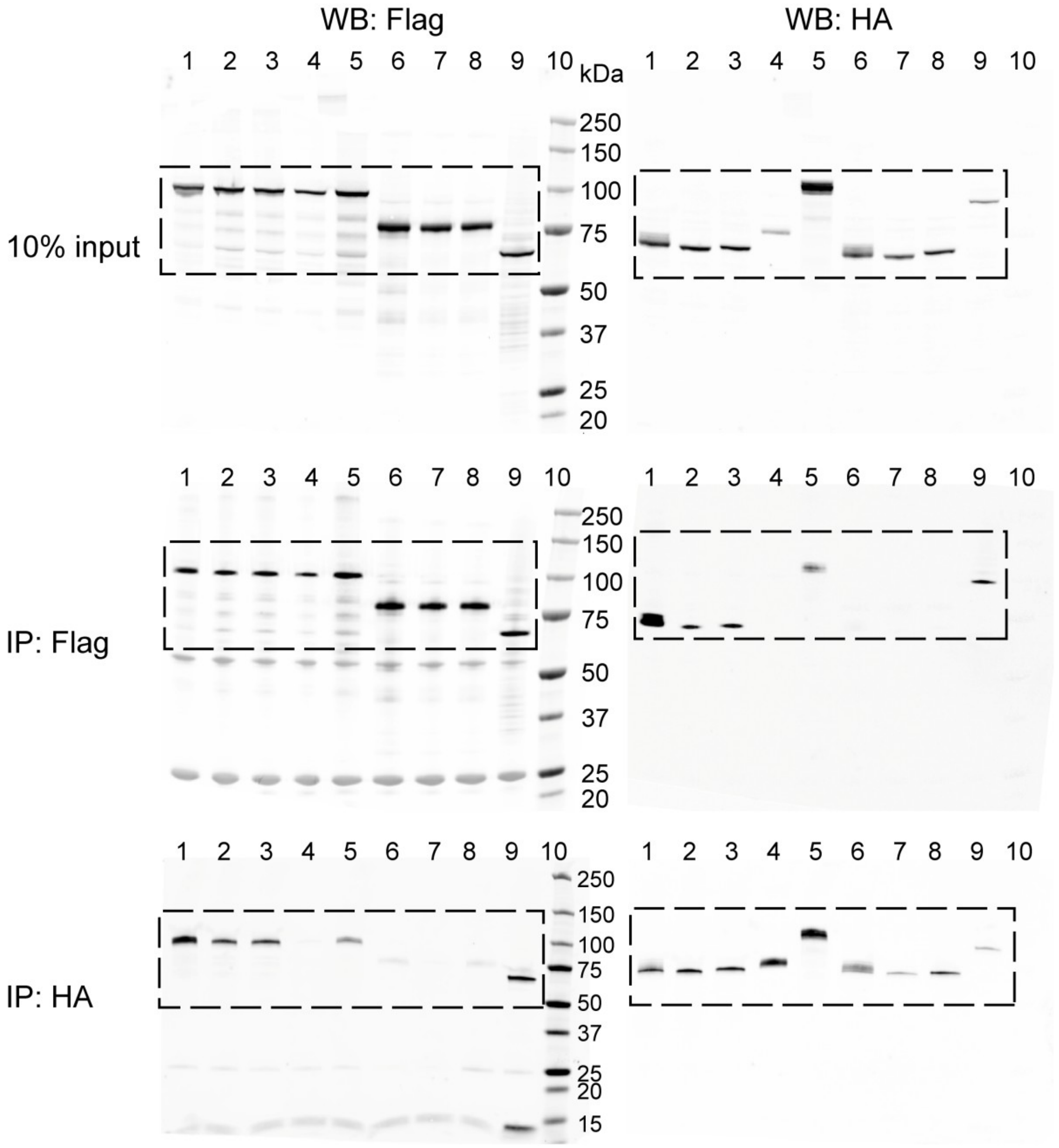

Figure S6. Western blots of coimmunoprecipitation of POC1B with FAM161A

Uncropped immunoblots of the coimmunoprecipitation assay of FAM161A and POC1B. Dashed rectangles indicate cropped immunoblots shown in Figure 5 and S5. The upper blots show 10% of the input used in the coimmunoprecipitation experiment, the middle blots the FLAG precipitates and the lower blots the HA precipitates. The protein bands were visualized by either staining with FLAG-antibody (left blots) or HA-antibody (right blots). Lane numbering as follows, lane 1: 3xFLAG-FAM161A + 3xHA-POC1B-WT; lane 2: 3xFLAG-FAM161A + 3xHA-POC1B-Gln67del; lane 3: 3xFLAG-FAM161A + 3xHA-POC1B-Arg106Pro; lane 4: 3xFLAG-FAM161A + 3xHA-p63; lane 5: 3xFLAG-FAM161A + 3xHA-lebercilin; lane 6: 3xFLAG-p63 + 3xHA-POC1B-WT; lane 7: 3xFLAG-p63 + 3xHA-POC1B-Gln67del; lane 8: 3xFLAG-p63 + 3xHA-POC1B-Arg106Pro; lane 9: 3xFLAG-nephrocystin-4 + 3xHA-RPGRIP1; lane 10: Marker. Size markers are depicted in kDa.

Figure S7. Localization studies of POC1B and FAM161A in hTERT-RPE1 cells

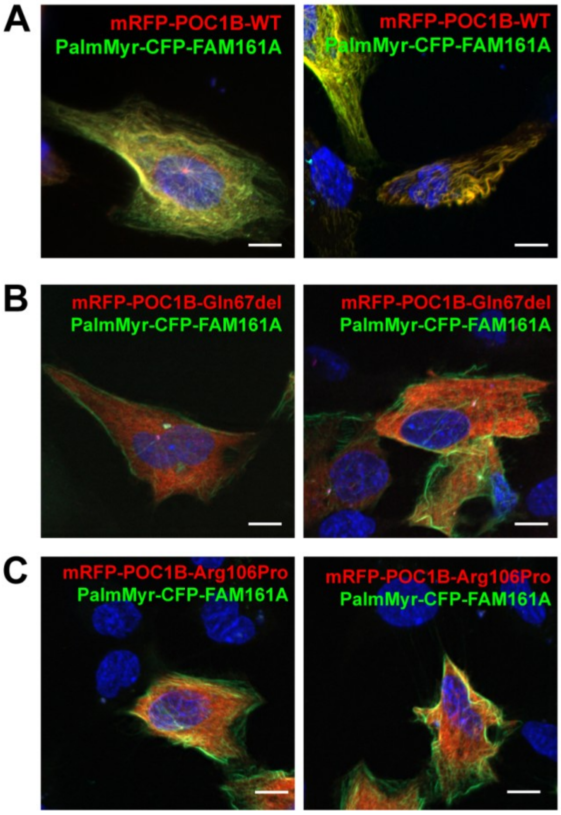

Figure S7. Localization studies of POC1B and FAM161A in hTERT-RPE1 cells

Overlay photomicrographs of additional fields of hTERT-RPE1 cells corresponding to the images depicted in Figure 5B-D. (A) Overlay images of localization upon overexpression in hTERT-RPE1 cells. PalmMyr-CFP-FAM161A (green) was targeted to the cell membrane and microtubules, and translocated mRFP-POC1B wild-type (red) from the cytosol towards the cell membrane and microtubules. (B) This translocation by PalmMyr-CFP-FAM161A (green) is not observed for mRFP-POC1B-Gln67del (red) nor (C) mRFP-POC1B-Arg106Pro (red) which both maintain their cytosolic localization. RPGRIP1L (cyan) was used as transition zone marker of the cilium. Nuclei are stained with DAPI (blue). Scale bars: 10 μm.

Table S1. Primer sequences for *POC1B*

POC1B exon	Forward	Reverse	Product size (bp)
Exon 1	tctccttccccatcctctc	gctacggacacctgcctc	205
Exon 2	gactccggggaagtggc	ccggcccatggagtttag	225
Exon 3	gccgctctattacctggatg	gagaataacagtgcaggcagc	286
Exon 4	catggaaatttcagtgttgacg	ttgtttactaccgttcccgc	519
Exon 5	catgggatggtaacagtggtc	cccggccatatttcaatttc	340
Exon 6	tgcggtgagctgacattg	cccatgaccccaagtaagac	338
Exon 7	ttgctaataaggcactgggg	agcagcaatgtgtgctatgtc	332
Exon 8	actctgccagcattagttgc	tcttagctgaggtcaaggattc	294
Exon 9	ttgtgggttttaaaatcagctc	cctccttcagcaaaagcctc	293
Exon 10	tgaaaaccttttatttctgggg	cctggaaattgttgctgctc	521
Exon 11	aaatcttcaatgtaagcctggg	agaaatctcccctgctctgc	434
Exon 12	gtcatcctcaccaccagagg	gcacatggttgtgttgtatgg	265

Table S2. Primer sequences for *POC1B* mRNA analysis

Gene	Forward	Reverse	Product size (bp)
POC1B	cacccgatggaagactaattg	aagaagatgtggtggtgaatc	521
GUSB	ctgtacacgacacccaccac	tacagataggcagggcgttc	245

Table S3. Variants after stringent filtering for family A

Gene	DNA	Protein	Coverage	% variation	SNP allele frequency	Allele frequency in house-database	PhyloP	Grantham	SNP identity
Homozygous									
POC1B	c.317G>C	p.Arg106Pro	50	98	0	0	6.13	103	rs76216585
Compound heterozygous									
ASTE1	c.95G>T	p.Gly32Val	112	46.4	0	0	5.32	109	
ASTE1	c.796T>G	p.Phe266Val	36	22.2	0	0	4.63	50	
CNTN3	c.725G>A	p.Gly242Asp	51	27.4	0.0221	0	2.54	94	rs150505018
CNTN3	c.2308C>T	p.Pro770Ser	98	27.5	0	0	2.15	74	
TUBGCP2	c.1064C>T	p.Thr355Met	8	37.5	0	0	5.55	81	
TUBGCP2	c.172C>T	p.Arg58Cys	21	38.1	0	0	2.20	180	

Table S4. Variants after stringent filtering for family B

Gene	DNA	Protein	Coverage	% variation	SNP allele frequency	Allele frequency in house-database	PhyloP	Grantham	SNP identity
Compound heterozygous									
NUDT14	c.154C>T	p.Arg52Trp	38	20	0.0665	0	0.96	101	rs142762135
NUDT14	c.136C>A	p.Leu46Ile	33	15	0.0667	0	3.99	5	rs148004115
PIKFYVE	c.1770C>T	p.=	50	17	0.1781	0.23	3.41	-	rs61752185
PIKFYVE	c.5726C>T	p.Ala1909Val	20	10	0	0	5.84	64	
POC1B	c.810+1G>T	p.Phe188Aspfs*73/p.Val226Glyfs*30	62	27	0	0.08	6.072	-	
POC1B	c.199_201del	p.Gln67del	27	6	0	0.15	4.68	-	
